# The dysregulation of biliary tract microflora is closely related to primary choledocholithiasis: a multicenter study

**DOI:** 10.1038/s41598-024-59737-6

**Published:** 2024-04-18

**Authors:** Min Xiao, Yankun Zhou, Zhengfei Wang, Wenchao Dai, Di Wang, Zhenmiao Wan, Zhitao Chen, Qiyong Li, ShuSen Zheng

**Affiliations:** 1 Zhejiang University School of Medicine, Hangzhou, Zhejiang China; 2 Department of Surgery, Shulan (Hangzhou) Hospital, Zhejiang Shuren University Shulan International Medical College, Hangzhou, Zhejiang China; 3 Department of Surgery, Quzhou People’s Hospital, Quzhou, Zhejiang China; 4 Department of Surgery, Shulan (Quzhou) Hospital, Quzhou, Zhejiang China; 5 Division of Hepatobiliary and Pancreatic Surgery, Zhejiang Chinese Medical University, Hangzhou, Zhejiang China; 6 Jinan Microecological Biomedicine Shandong Laboratory, Jinan, Shandong China

**Keywords:** Bile microbiota, Choledocholithiasis, Gallbladder polyp, Actinobacteriota, Enterococcus, Ecology, Immunology, Risk factors

## Abstract

Bile microecology changes play an important role in the occurrence and development of choledocholithiasis. At present, there is no clear report on the difference of bile microecology between asymptomatic patients with gallbladder polyps and choledocholithiasis. This study compared bile microecology between gallbladder polyp patients and patients with choledocholithiasis to identify risk factors for primary choledocholithiasis. This study was conducted in 3 hospitals in different regions of China. Bile samples from 26 patients with gallbladder polyps and 31 patients with choledocholithiasis were collected by laparoscopic cholecystectomy and endoscopic retrograde choledocholithiasis cholangiography (ERCP), respectively. The collected samples were used for 16S ribosomal RNA sequencing and liquid chromatography mass spectrometry analysis. The α-diversity of bile microecological colonies was similar between gallbladder polyp and choledocholithiasis, but the β-diversity was different. Firmicutes, Proteobacteri, Bacteroidota and Actinobacteriota are the most common phyla in the gallbladder polyp group and choledocholithiasis group. However, compared with the gallbladder polyp patients, the abundance of *Actinobacteriota* has significantly lower in the choledocholithiasis group. At the genera level, the abundance of a variety of bacteria varies between the two groups, and *Enterococcus* was significantly elevated in choledocholithiasis group. In addition, bile biofilm formation—*Pseudomonas aeruginosa* was more metabolically active in the choledocholithiasis group, which was closely related to stone formation. The analysis of metabolites showed that a variety of metabolites decreased in the choledocholithiasis group, and the concentration of beta-muricholic acid decreased most significantly. For the first time, our study compared the bile of gallbladder polyp patients with patients with choledocholithiasis, and suggested that the change in the abundance of Actinobacteriota and Enterococcus were closely related to choledocholithiasis. The role of *Pseudomonas aeruginosa* biofilm in the formation of choledocholithiasis was discovered for the first time, and some prevention schemes for choledocholithiasis were discussed, which has important biological and medical significance.

## Introduction

Choledocholithiasis is a chronic recurrent disease of the liver and the gallbladder, and is one of the most common diseases of the digestive system. In the West, most common bile duct stones form in the gallbladder, and less than 10% of common bile duct stones reform in the common bile duct. In the East, the incidence of primary choledocholithiasis is much higher than in the West^1^. To date, many studies have shown that bacterial infection is closely related to the onset of choledocholithiasis. Maluenda et al. found many bacteria in the bile or stone samples of patients with cholelithiasis, including *Escherichia coli*, *Klebsiella pneumoniae*, *Enterococcus faecium* and *Enterococcus faecalis*^2^. The classic bile pigment-calcium stone formation hypothesis proposed by Maki, which is widely accepted, is that bacterial enzymes hydrolyzing bilirubin, such as β-glucuronidase and phospholipase, promote stone formation^3^. In addition, continuous bacterial infection can activate the immune response of the bile duct epithelial cells, secreting a large number of inflammatory cytokines, such as IL-6 and IL-10, and further promote the accumulation of bacteria to cause stone formation^4^. Multidrug-resistance (MDR) efflorescent pump proteins expressed by bacteria can produce bile resistance, allowing bacteria to survive in certain ecological environments along with bile salts^5,6^. It has been shown that the elimination of pathogens in certain bile ducts can reduce the incidence of gallstones.

To date, the study of bile microecology in patients with choledocholithiasis has mainly focused on the comparison of bile duct and duodenal microecology. Han and Lyu have suggested that biliary flora in patients with choledocholithiasis are similar in composition to duodenal fluid flora, and proposed the hypothesis that duodenal-bile reflux plays an important role in possible stone formation^7,8^. However, these studies ignored the effect of primary changes in biliary microflora on stone formation, so it is important to compare the difference in bile microecology between healthy people and patients with choledocholithiasis. Because of the ethical problems associated with obtaining bile from a healthy population, we used bile from patients with asymptomatic gallbladder polyps to maximize the simulation of bile from a healthy population.

Due to the coexistence of multiple bacteria in the biliary tract, the culture-dependent method is not sensitive to bacterial identification and is not sufficient to study the entire microbiome^9,10^. High-throughput sequencing techniques have revealed the composition of microorganisms and facilitated the discovery of new biliary bacteria, which can enrich our understanding of the microecology of the biliary tract. In this study, high-throughput 16S ribosomal RNA (16S rRNA) gene sequencing and liquid chromatography mass spectrometry (LC–MS) were used to compare the bacterial communities and metabolomics in bile from gallbladder polyp patients and patients with choledocholithiasis, in order to investigate the following three aspects: (1) The structure and function of the bile microbial community in the gallbladder polyp patients; (2) the structure and function of the bile microbial community in patients with choledocholithiasis; (3) and a comparison of the different flora in the bile of gallbladder polyp patients and patients with choledocholithiasis and predict their role in the occurrence and development of the diseases.

## Material and methods

### Study design and sample collection

This study was conducted in 3 hospitals in China, namely Shulan (Hangzhou) Hospital, Shulan (Quzhou) Hospital and Quzhou People’s Hospital. This study recruited patients with gallbladder polyps and choledocholithiasis between May 2022 and April 2023.

We replaced healthy people with asymptomatic, non-infectious patients with gallbladder polyps. The following inclusion criteria were used for patients with gallbladder polyps: (1) Patients diagnosed with gallbladder polyps due to clinical symptoms and signs imaging examination; (2) patients who had a laparoscopic cholecystectomy performed in our hospital where the postoperative pathology was gallbladder polyp; and (3) patients who had not taken antibiotics or probiotics for seven days prior to surgery (except 30 min before surgery). The exclusion criteria were as follows: (1) Patients with cholecystolithiasis, choledocholithiasis or other hepatobiliary pancreatic diseases; (2) patients with biliary tract injury during surgery, or with a history of biliary tract surgery and digestive tract diversion surgery before surgery; and (3) patients with heart, brain, kidney or other major diseases, or who, for other reasons, researchers believed not to be suitable to participate in the test.

The inclusion criteria for patients with choledocholithiasis were as follows: (1) Patients with primary choledocholithiasis who had been diagnosed by preoperative imaging examination based on clinical symptoms and signs; (2) patients with no previous history of endoscopic retrograde cholangiography or bile duct exploration; and (3) patients who had not taken antibiotics or probiotics for seven days prior to surgery (except 30 min before surgery). The exclusion criteria were as follows: (1) Patients with acute cholangitis, gallstones or other hepatobiliary pancreatic diseases; (2) patients with a preoperative history of biliary tract surgery or digestive tract diversion surgery; and (3) patients with heart, brain, kidney or other major diseases, or who, for other reasons, researchers believed not to be suitable for participating in the test.

Bile was obtained from patients with choledocholithiasis through endoscopic retrograde cholangiopancreatography (ERCP), which was performed by experienced doctors in a dedicated operating room, and the entire procedure was strictly aseptic. Strict sterilization was ensured before duodenoscopic surgery to keep the working channels sterile. In the process of putting endoscope from mouth into duodenum before suction of bile, the work channel of endoscope kept itself clean by avoiding pumping action. After Oddi sphincterotomy, 20 ml sterile saline was used to flush the lateral endoscopic tube, and bile samples were collected immediately through a sterile catheter. An amount of 3–5 mL bile was extracted, and the samples were placed in sterile test tubes and immediately stored in a refrigerator at − 80 ℃. Bile was obtained surgically from patients with gallbladder polyps. The gallbladder was obtained by laparoscopic cholecystectomy. An amount of 3–5 ml bile was extracted in a sterile manner from the removed gallbladder. The bile sample was stored in a refrigerator at − 80 °C until used for DNA extraction.

We collected a total of 57 bile samples, 26 from the gallbladder polyp group and 31 from the choledocholithiasis group. Clinical data were collected for each patient, including age, sex, body mass index (BMI), blood routine, liver and kidney function. All patients in this study expressed understanding and signed informed consent, and the study was approved by the Ethics Committee of Shulan (Hangzhou) Hospital.

### DNA extraction, illumina MiSeq sequencing and bioinformatic analysis

The bacterial DNA was isolated from bile samples using the MagPure Soil DNA LQ kit (Magen, Guangdong, China), DNA integrity and size were assessed by 1% agarose gel electrophoresis, and DNA concentration and quality were tested by a NanoDrop 2000 spectrophotometer (Thermo Fisher Scientific, Waltham, MA, USA). PCR amplification of the V3–V4 hypervariable regions of the bacterial 16S rRNA gene was using universal primer pairs (343F: 5′-TACGGRAGGCAGCAG-3′; 798R:5′-AGGGTATCTAATCCT-3′).

Amplicon quality by gel electrophoresis. The PCR products were purified with Agencourt AMPure XP beads (Beckman Coulter Co, USA) and quantified using Qubit dsDNA assay kit (Life Technologies, California, USA). Then, sequencing was performed on an Illumina NovaSeq6000 with 250 bases per sequencing cycle (Illumina Inc., San Diego, CA; OE Biotech Company; Shanghai, China)^11^. Remove raw sequencing reads without V3–V4 primers using Cutadapt. To detect the high-quality sequences, denoised with the DADA2 protocol. This method replaces clustering methods based on sequence similarity and obtains reliable amplicon sequence variation (ASV) by correcting sequence errors through de-duplication, chimera filtering and low sequence removal. Selection of representative readings for each ASV using the QIIME2 software package. All representative reads were annotated using the q2-feature-classifier with default parameters and compared to the Silva database Version 138^12,13^.

All statistical analyses were conducted using the R programming language. The Abundance based Coverage Estimator (ACE) index, Chao1 index, observed species (OBS) index, Shannon index and Simpson index were used to reflect the α-diversity of species. The microecological differences between different samples can be judged by β-diversity and evaluated by principal coordinate analysis (PcoA). PcoA is calculated by Binary_Jaccard distance algorithm, Weighted Unifrac distance algorithm and Unweighted Unifrac distance algorithm. A linear discriminant analysis (LDA) effective size (LEfSe) algorithm was introduced to identify the features most likely to explain differences between gallbladder polyp and choledocholithiasis microbiota by LDA score greater than or equal to 2. PICRUSt was used to the predict bacterial function through the Kyoto Encyclopedia of Genes and Genomes (KEGG) database^14–16^. The Wilcoxon rank sum test was used to evaluate differences between populations. A *p* < 0.05 was required for the results to be considered statistically significant.

### Metabolomics analysis

Quantitative analysis of bile metabolic components by high performance liquid chromatography-mass spectrometry (LC–MS). The bile samples stored at − 80 °C were thawed at room temperature. Methanol and water were added to each sample. Chloroform was added to each aliquot. Cells were broken using an ultrasonic homogenizer at 500 w for 6 min. All mixtures of each sample were transferred to a 1.5 mL Eppendorf tube. l-2-Chlorophenylalanine was dissolved in methanol to serve as an internal standard. The samples were centrifuged at 4 °C (13,000 rpm) for 10 min. The supernatant of each tube was collected with a crystallization syringe, filtered through a 0.22 μm microfilter and transferred to an LC vial.

ACQUITY UPLC I-Class system (Waters Corporation, Milford, USA) coupled with VION IMS QTOF Mass spectrometer( Waters Corporation, Milford, USA) was used to analyze the metabolic profiling in ESI-positive and ESI-negative ion modes. An ACQUITY UPLC BEH C18 column (1.7 μm, 2.1 × 100 mm) were employed in both positive and negative modes. The mobile phase consisted of solvent A (water, 0.1% formic acid) and solvent B (acetonitrile, 0.1% formic acid). The linear gradient: 0 min, 1% B; 1 min, 30% B; 2.5 min, 60% B; 6.5 min, 90% B; 8.5 min, 100% B; 10.7 min, 100% B; 10.8 min, 1% B and 13 min, 1%B. The flow rate was 0.4 mL/min and column temperature were 45°℃. Data acquisition was performed in full scan mode (m/z ranges from 50 to 1000). The mass spectrometry parameters were: low-energy scan (CE 4 eV), and and high-energy scan (CE ramp 20–45 eV) for fragmentation of ions. The collision-induced dissociation gas was argon, and the scan time was 0.2 s; inter-scan delay: 0.02 s; capillary voltage: 2.5 kV; cone voltage: 40 V; source temperature: 115 °C; desolvation gas temperature: 450 °C; and desolvation gas flow: 900 L/h^17^. The original LC–MS data were processed by software Progenesis QI V2.3 (Nonlinear, Dynamics, Newcastle, UK) for baseline filtering, peak identification, integral, retention time correction, peak alignment, and normalization.

The matrix was imported in R programming and Principal Component Analysis (PCA) was performed to observe the overall distribution among the samples and the stability of the whole analysis process. Partial Least-Squares-Discriminant Analysis (PLS-DA) was utilized to distinguish the metabolites that differ between two groups. To prevent overfitting, sevenfold cross-validation and 200 Response Permutation Testing (RPT) were used to assess the quality of the model. Differential metabolites were selected with *p*-values less than 0.05.

### Data analysis

The normality of the data was tested using the Kolmogorov–Smirnov or Shapiro–Wilk tests. Continuous variables with a normal distribution are presented as mean ± standard deviation (SD), and non-normal variables are reported as median (interquartile range). Categorical variables are presented as percentages. In terms of the normal distribution and homogeneity of variance, statistical significance was tested using the student’s *t*-test, Welch’s *t*-test, or the non-parametric Wilcoxon rank sum test. SPSS software (Version 27.0, IBM SPSS Inc., Chicago, USA) was used for the analyses. Two-tailed *p* < 0.05 was considered as indicating a statistically significant difference.

### Institutional review board statement

This study was conducted according to the guidelines of the Declaration of Helsinki and approved by the Ethics Committee of Shulan (Hangzhou) Hospital (KY2021067).

## Results

### Patients’ characteristics

Between May 2022 and April 2023, a total of 57 patients were recruited for the study, namely, 26 patients (9 males and 17 females) for the gallbladder polyp group and 31 patients (12 males and 19 females) for the choledocholithiasis group. Blood test results were collected from all patients the day before the bile specimens were obtained. The characteristics of the patients are summarized in Table 1. There were significant differences in baseline characteristics for age, CRP, ALB, GLB, ALT, AST, GGT, ALP, and TBIL. Gallbladder polyps usually develop in middle-aged patients around 50 years old^18^, while the incidence of choledocholithiasis increases with age and is more common in elderly patients. Choledocholithiasis can cause transient cholestasis, which can lead to abnormal liver function and inflammatory markers, with elevated ALP being the most common liver function abnormality^19^.

**Table 1:** Clinical patient characteristics.

	Gallbladder polyp group	Choledocholithiasis group	p-value
Patients, n	26	29	NA
Male, n%	9 (34.6%)	12 (41.3%)	1
Age (years)	48.6 ± 11.3	65.1 ± 14.3	0.001
Heigh (m)	1.6 ± 0.07	1.6 ± 0.07	0.608
Weight (kg)	66.1 ± 10.3	60.8 ± 9.1	0.055
BMI (kg/m^2^)	24.8 ± 2.7	23.2 ± 3.1	0.053
WBC (10–9/L)	5.8 (4.2–6.6)	6.3 (4.7–7.7)	0.443
HB (g/L)	134.6 ± 20.5	129.7 ± 19.5	0.378
Blood Plt (10E9/L)	214.1 ± 46.9	214.3 ± 75.0	0.989
CRP (mg/L)	5.2 ± 2.1	38.8 ± 69.9	0.003
ALB (g/L)	44.3 ± 3.1	40.9 ± 4.6	0.003
Glb (g/L)	26.5 ± 4.2	30.6 ± 4.4	0.028
ALT (U/L)	18.5 (12.2–34)	30 (16–259)	0.017
AST (U/L)	21.6 (19–26.6)	34 (21–267)	0.005
GGT (U/L)	18.5 (15.1–22.5)	136 (41–396)	< 0.001
ALP (U/L)	66.5 (61.2–80.7)	121 (83–174)	< 0.001
TBIL(umol/L)	10.9 (8.4–15.3)	20 (14–52)	< 0.001
Cr (umol/L)	61 (52.3–77.2)	74 (63–79)	0.121
TC (mmol/L)	4.7 ± 1.1	4 ± 0.8	0.084
TG (mmol/L)	1.4 ± 0.7	1.3 ± 0.6	0.815
HDL (mmol/L)	1.2 ± 0.3	1 ± 0.4	0.281
LDL (mmol/L)	2.7 ± 0.7	2.2 ± 0.8	0.124
INR	0.96 (0.93–1)	0.99 (0.95–1.03)	0.213

Data are expressed as percentages for categorical variables and as mean (SD) for continuous variables in the case of normal distributions and as median (lQR) otherwise.

BMI: body mass index; WBC: white blood cell; Hb: hemoglobin; Plt: platelet; CRP: C-reactive protein; ALB: albumin; GLB: globulin; ALT: alanine aminotransferase; AST: aspartate aminotransferase; GGT: gamma-glutamyl transpeptidase; ALP: alkaline phosphatase; TBIL: total bilirubin; Cr: creatinine; TC: total cholesterol; TG: total triglycerides; HDL: high density lipoprotein; LDL: low-density lipoprotein; INR: international normalized ratio.

### Sequencing data and bacterial diversity analysis

A total of 57 samples were submitted for 16S rRNA sequencing. The volume of raw data after sequencing ranged from 78,001 to 81,933, and the volume of clean tags data after quality control ranged from 67,166 to 77,618. After removing chimeras from the clean tags, the valid tags data ranged from 46,149 to 74,994. The number of ASVs in each sample ranged from 52 to 802. A total of 3887 differential ASVs were identified. Good’s coverage (%) of all groups was above 99%, that is, the sequencing amount of each sample is close to saturation. Therefore, this study has high reliability and reference value.

**Figure 1:**
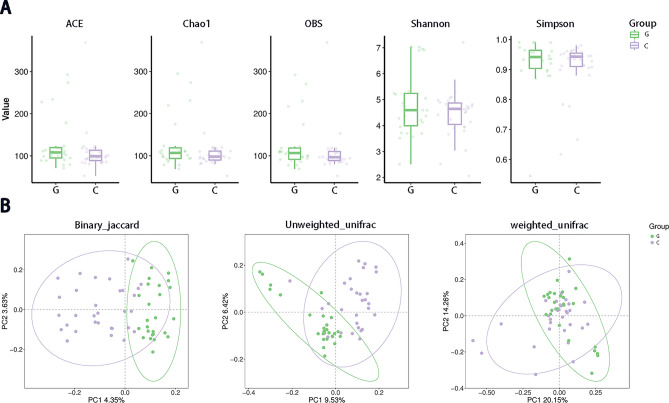
(**A**) The α-diversity between the two groups was calculated using five different parameters: ACE, Chao1, OBS, Shannon, and Simpson. (**B**) The β-diversity was calculated using the binary_jaccard distance, the unweighted UniFrac distance, and the weighted UniFrac distance. G: gallbladder polyp group; C: choledocholithiasis group.

Chao1, ACE, OBS, Simpson, and Shannon diversity indexes were used to reflect α-diversity. The sequencing curve of each sample tends to be horizontal, indicating that sufficient sequencing depth has been achieved to cover most of the microbial information in the sample. Through the analysis of α-diversity of the gallbladder polyp group and the choledocholithiasis group, it was found that the ACE, Chao1, and OBS indexes for calculating the bacterial abundance of the two groups were not significantly different (*p* > 0.05). There was no significant difference between the Shannon index and the Simpson index in calculating the bacterial distribution evenness index (*p* > 0.05). This indicated that the abundance of microbiota was similar for the two groups (Fig. 1A).

A principal coordinate analysis (PCoA) was used to analyze the β-diversity of the microbial communities in the two groups. According to the Binary_jaccard distance algorithm, the samples of the gallbladder polyp group were on the left side of the vertical axis and the samples of the choledocholithiasis group were on the right side. The unweighted_UniFrac distance algorithm yielded similar results. The weighted_UniFrac distance algorithm took into account the abundance of species, so the sample distribution is more concentrated. In conclusion, the microbiota composition of the gallbladder polyp group and that of the choledocholithiasis group are different (binary_jaccard, *p* = 0.01; unweighted UniFrac, *p* = 0.01; weighted UniFrac, *p* = 0.15) (Fig. 1B).

### Analyses of the microbial community structures in the two groups

**Figure 2:**
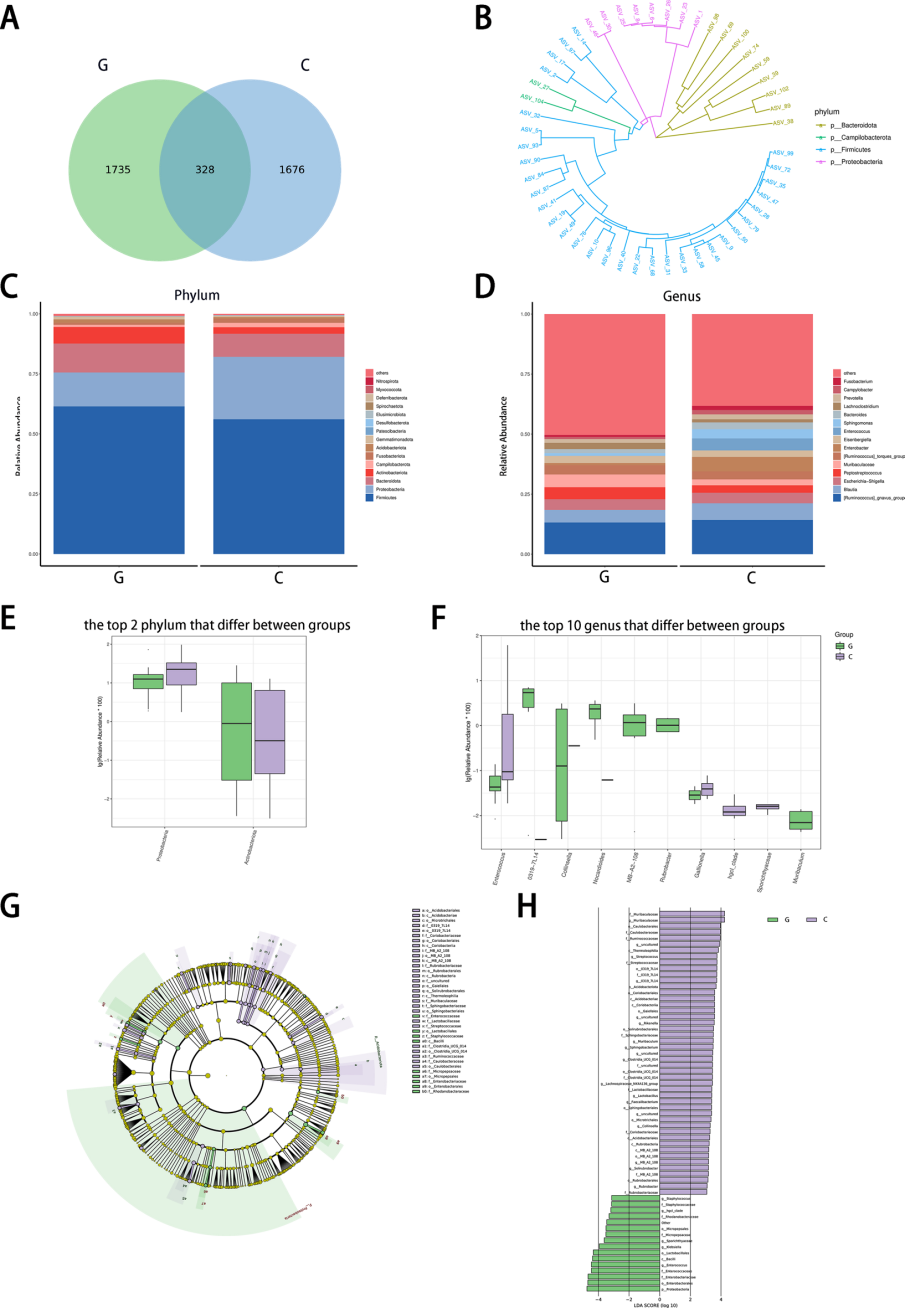
(**A**) Venn diagram. Venn diagram of ASVs indicating the number of common and unique ASVs, and the similarity and overlap of ASVs between the two groups. (**B**) The evolutionary tree shows the phyla of the 50 most abundant ASVs. (**C**) The difference between the two groups at the phylum level. (**D**) The difference of the two groups at the genus level. (**E**) Differential phyla were detected between the two groups. For optimal visualization, a transformation of log10 (relative abundance × 100) was employed. (**F**) The top 10 differential genera that were detected between the two groups. (**G**) LEfSe differential species annotation branch diagram. Different colors represent different groups, each node on a different classification grade represents a classification of that grade, and the diameter of the small circle is proportional to the relative abundance. Each layer node represents the phylum/class/order/family/genus from the inside out. (**H**) The histogram of the LEfSe analysis revealed bacteria whose LDA scores exceeded the default value of 2. G: gallbladder polyp group; C: choledocholithiasis group.

Only 328 ASVs were co-present in the gallbladder polyp group and the choledocholithiasis group, representing 10.6% of all ASVs (see Fig. 2A). The top 50 most abundant ASVs are the Firmicutes, *Bacteroidota*, Proteobacteria and Campilobacterota, respectively (Fig. 2B).

At the phylum level, a total of 21 phyla were identified in the two groups. The most common phyla in the gallbladder polyp group were Firmicutes (61.9%), followed by Proteobacteria (13.9%), *Bacteroidota* (11.6%), and *Actinobacteriota* (6.9%). The most common phyla in the choledocholithiasis group were Firmicutes (56.1%), followed by Proteobacteria (25.7%), *Bacteroidota* (9.9%), and *Actinobacteriota* (2.6%) (Fig. 2C). We found that the abundance of Proteobacteria (*p* = 0.02) and *Actinobacteriota* (*p* = 0.01) were significantly different in the distribution of gallbladder polyp group and choledocholithiasis group. The abundance of *Actinobacteriota* in the gallbladder polyp group was high, while that of Proteobacteria was low (Fig. 2E).

At the genera level, 232 genera were identified in the two groups. In the gallbladder polyp group, the most common genera were *Ruminococcus* (13%), *Blautia* (5.3%), and *Muribaculaceae* (5.2%). The most common genera in the choledocholithiasis group were *Ruminococcus* (14.1%), *Blauti* (6.9%), and *Enterococcus* (5.1%) (Fig. 2D). The 10 genera with significant differences were *Enterococcus* (*p* = 0.01), 0319-7L14 (*p* = 0.01), *Collinsella* (*p* = 0.03), *Nocardioides* (*p* = 0.04), MB-A2-108 (*p* = 0.02), *Rubrobacter* (p = 0.03), *Gallionella* (*p* = 0.001), *hgcI_clade* (*p* = 0.002), *Sporichthyaceae* (*p* = 0.03) and *Muribaculum* (*p* = 0.02). It is worth noting that, except for *Enterococcus*, *Gallionella*, *hgcI_clade*, and *Sporichthyaceae*, the abundance of other genera in the gallbladder polyp group was higher than that in the choledocholithiasis group (Fig. 2F).

The LEfSe analysis results also indicate that there were significant differences in the abundance of microorganisms in class, order, family, and genus between the gallbladder polyp group and the choledocholithiasis group (Fig. 2G,H).

### Prediction of microbial metabolic function

**Figure 3:**
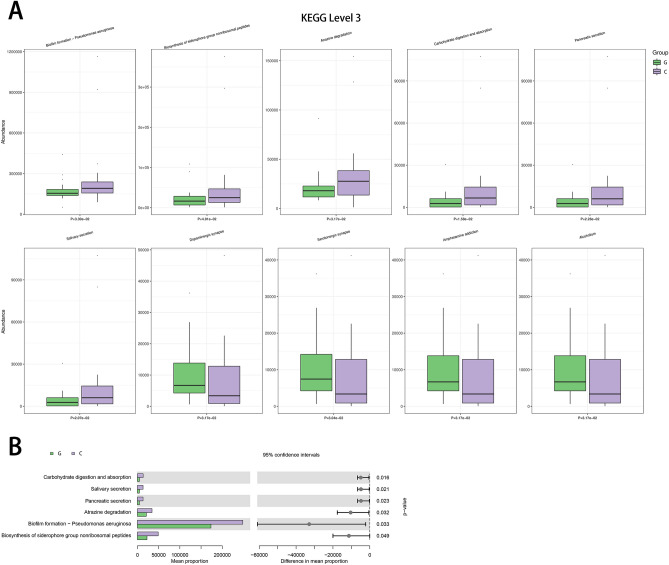
Prediction of microbial function in bile samples. (**A**) The 10 most significantly enriched pathways were found at KEGG Level 3. (**B**) Six pathways were significantly enriched in the choledocholithiasis group. G: gallbladder polyp group; C: choledocholithiasis group.

In order to gain a deeper understanding of the metabolic function disparities among microbial communities, we conducted functional prediction analysis using the Kyoto Encyclopedia of Genes and Genomes (KEGG) database. At KEGG level 3, we identified 360 metabolic pathways. Of the ten metabolic pathways with the most significant differences, six were more enriched in the choledocholithiasis group, namely biofilm formation—*Pseudomonas aeruginosa* (*p* = 0.033), biosynthesis of the siderophore group non-ribosomal peptides (*p* = 0.049), atrazine degradation (*p* = 0.032), carbohydrate digestion and absorption (*p* = 0.016), pancreatic secretion (*p* = 0.023) and salivary secretion (*p* = 0.021). Conversely, four pathways showed higher abundance in the gallbladder polyp group: dopaminergic synapse (*p* = 0.04), serotonergic synapse (*p* = 0.06), amphetamine addiction (*p* = 0.04), and alcoholism (*p* = 0.03) (Fig. 3A,B).

### Bile acid metabolomics analysis

**Figure 4:**
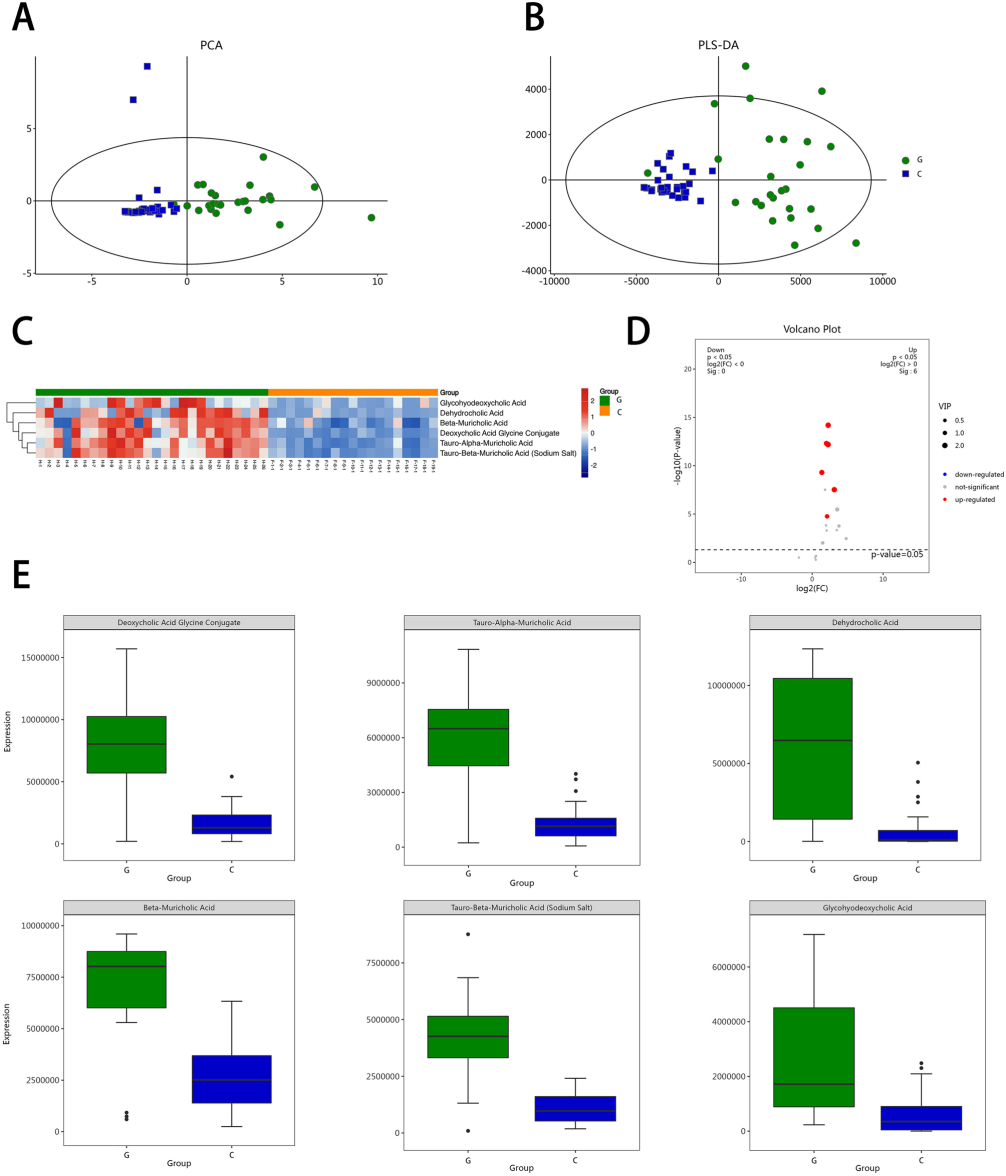
The metabolites of bile were analyzed. (**A**) Principal component analysis (PCA) is a kind of unsupervised analysis. The projected score value of each sample on the plane composed of the first principal component and the second principal component is the spatial coordinate, which can directly reflect the similarity or difference between samples. The ellipse represents a 95% confidence interval. (**B**) Partial least squares discrimination analysis (PLS-DA) is a supervised discriminant statistical method in which the horizontal coordinate represents the difference between groups. (**C**) Heatmaps of different metabolites between the two groups. (**D**) Volcano maps of different metabolites between the two groups. (**E**) Boxchart of differential metabolic pathways.G: gallbladder polyp group; C: choledocholithiasis group.

In order to understand the differences in metabolites between the two groups of samples, metabolites were analyzed using the method of metabolomics. The sample model was constructed using principal component analysis (PCA) and partial least squares discriminant analysis (PLS-DA). In both models, the abscissa is the predicted principal component, representing the difference between the groups, and the ordinate is the orthogonal principal component, representing the difference within a group (Fig. 4A,B).

We found six different metabolites, namely, glycohyodeoxycholic acid (p < 0.01), dehydrocholic acid (p < 0.01), beta-muricholic acid (p < 0.01), deoxycholic acid glycine conjugate (p < 0.01), tauro-alpha-muricholic acid (p < 0.01) and tauro-beta-muricholic acid (sodium salt) (p < 0.01) (Fig. 4C). These six metabolites were all enriched in the gallbladder polyp group (Fig. 4D,E).

## Discussion

In recent years, 16S rRNA gene sequencing is increasingly being applied to the study of the human microbiome, so that people have a more comprehensive and in-depth understanding of the human microecology. Bacteria have been shown to play an important role in the development of many diseases. Current studies on intestinal microecology provide a new perspective for exploring the pathogenesis of diseases. For example, intestinal floras are closely related to obesity, inflammatory bowel disease, and colorectal malignancy^20^. The onset and progression of choledocholithiasis are likewise closely linked to bacteria. To date, studies have primarily compared the microecology of bile in choledocholithiasis to that in duodenal microecology, leaving the distinction between bile microecology in healthy individuals and choledocholithiasis patients unclear.

The gallbladder is anatomically connected to the common bile duct via the gallbladder duct. It stores bile produced by the liver cells and releases it into the common bile duct through contractions. At the same time, bile in the common bile duct can flow back into the gallbladder, facilitating bile exchange between the two compartments^21^. Gallbladder polyps is a benign proliferative disease^22^. To date, no studies have established a connection between gallbladder polyps and changes in bile microecology. Additionally, Zhang et al. found no association between Helicobacter pylori infection and gallbladder polyps^23^. We believe that the bile microecology of patients with asymptomatic gallbladder polyps is basically the same as that of healthy people. Due to the ethical issues of collecting bile from normal healthy people we used bile from patients with asymptomatic gallbladder polyps to replace bile from healthy people. Through microecological analysis and metabolomics, 16s rRNA and LC–MS were for the first time used to investigate the difference in biliary microecology between gallbladder polyp patients and patients with choledocholithiasis. The specific flora associated with choledocholithiasis were investigated, and the metabolic pathways and metabolites associated with these floras were studied.

This study included more cases for analysis, which is helpful for further comparison of the difference in biliary microecology between gallbladder polyp patients and patients with choledocholithiasis. The number of identified ASVs indicated that bacteria were abundant in the bile of both the gallbladder polyp group and the choledocholithiasis group. We observed that the α-diversity was similar for the gallbladder polyp group and the choledocholithiasis group, but there was a significant difference in β-diversity, indicating that the microbial communities between the two groups were different. The results of analyzing the microbial species indicated that Firmicites was the most dominant phylum in the bile of the gallbladder polyp group and the choledocholithiasis group, while other phyla with high abundance included Proteobacteria, *Bacteroidota*, and *Actinobacteriota*. This is similar to the results of some previous studies^24^. Previous studies have shown Proteobacteria to be elevated in the bile fluid of patients with choledocholithiasis, which is the same as our results^7^. Proteobacteria can affect the metabolism of bile acids in the intestine. We speculate that stone formation is influenced through the metabolism of bile acids^25^. We found a higher abundance of *Actinobacteriota* in the gallbladder polyp group. *Actinobacteriota* can produce short chain fatty acids (SCFAs) from carbohydrate fermentation, such as acetate, propionate, and butyrate, providing energy for the renewal of epithelial cells and their powerful antibacterial activity^26,27^. Butyrate is particularly important in the fight against bacterial toxins^28^. In addition, *Actinobacteriota* can also regulate inflammation and the immune response by inducing regulatory t cells, and chronic inflammation is an important cause of common bile duct stones^29,30^. In the choledocholithiasis group, the abundance of *Actinobacteriota* decreased, and the immune barrier and inflammatory regulation functions of the biliary tract were impaired, forming a microenvironment conducive to stone formation. The changes in the distribution of Proteobacteria and *Actinobacteriota* seem to be the major difference at the phylum level between the gallbladder polyp group and the choledocholithiasis group.

The most dominant genus at the generic level is the *Ruminococcus_gnavus*_group. Natalia et al. isolated *Ruminococcus* from the bile of donors without liver and bile disease^31^, and according to the bacterial distribution map, there is no significant difference in the distribution and abundance of *Ruminococcus* in the gallbladder polyp group and the choledocholithiasis group. We guess that *Ruminococcus* exists as a normal bacterial group in bile. We noted that the abundance of *Enterococcus* and *Gallionella* was significantly increased in the choledocholithiasis group. *Enterococcus* is a common infecting organism in acute cholangitis and malignant biliary obstruction^32,33^. *Enterococcus* can produce a pheromone-induced surface protein, which is involved in extracellular matrix adhesion and phagocytosis protection in order to improve its survival ability in the biliary tract^34,35^. *Enterococcus* is resistant to almost all cephalosporins, clindamycin and aminoglycoside antibiotics^36^, a fact which deserves our attention. In the treatment of patients with choledocholithiasis accompanied by cholangitis, antibiotics should be selected with more caution. In addition, *Enterococcus* produces large amounts of β-glucuronidase^37^. This enzyme is required for the deconjugation of bilirubin diglucuronide, and free bilirubin precipitates with Ca^2+^ to form calcium bilirubinate, the major component of brown pigment stones^38^. Therefore, we believe the abnormal variation of *Enterococcus* abundance can be a potential marker for predicting the development of choledocholithiasis. *Gallionella* is usually isolated from natural soils and is closely related to iron oxidation^39^. However, its role in the occurrence and development of choledocholithiasis remains unclear, and further studies are needed to explore its influence.

We also found that the abundance of other bacteria genera in the choledocholithiasis group was significantly lower than those in the gallbladder polyp group. Notably, increasing evidence has highlighted that microbiota can mediate gastrointestinal cancers and chronic diseases by microbial translocation, immunomodulation, metabolism, and enzymatic degradation and reduce ecological diversity^40–42^. Therefore, the reduction of bile microecological diversity can promote stone formation.

Microbial communities can change their microenvironment by forming metabolites. Bacteria have the potential to carry out thousands more chemical reactions than humans, with metabolic capabilities that far exceed our own^43^. Among the 10 KEGG pathways with significant differences, six were more enriched in choledocholithiasis, among which biofilm formation of *Pseudomonas aeruginosa* was the most significant. *Pseudomonas aeruginosa* metabolically produces several exopolysaccharides, including alginate, Psl, Pel, and lipopolysaccharide (LPS), which are important components of biofilms^44^. The biofilm forms a protective extracellular matrix and envelops the bacterial community within it, making the bacteria highly resistant to host immune clearance, antibiotics, and mechanical stress^45^. The bacteria fixed in the biofilm have a high survival rate and durable potential infectivity, and become the “potential flora” of biliary tract infection, which keeps the biliary tract in a chronic state of inflammation and is conducive to the formation of stones. Valdez found in mouse experiments that the probiotic *L. plantarum* has the ability to inhibit *Pseudomonas aeruginosa* biofilm^46^. The effect of *L. plantarum* in the human body is worth studying, and it can be a scheme to prevent the occurrence of common bile duct stones in patients with chronic cholangitis. Lyu et al. found a high abundance of *Pseudomonas aeruginosa* in the bile of duodenal fluid and choledocholithiasis patients^8^, but in our experiment, the abundance of *Pseudomonas aeruginosa* was not significant, which may be caused by the different locations of bile samples obtained (near the Oddi sphincter and near the stone). Low abundance *Pseudomonas aeruginosa* showed significant differences in metabolic pathways, which deserves our attention. Interestingly, the other five elevated metabolic pathways (carbohydrate digestion and absorption, salivary secretion, pancreatic secretion, atrazine degradation, and biosynthesis of siderophore group nonribosomal peptides) in the choledocholithiasis group are related to *Actinobacteriota*^47–51^. We hypothesized that these five metabolic pathways promote the generation of stones, and the high abundance of *Actinobacteriota* can inhibit these metabolic pathways. It has further been proved that a decreasing abundance of *Actinobacteriota* plays an important role in stone occurrence. In addition, we noted that alcohol metabolism was more active in the gallbladder polyp group. Kummerow reported that alcohol is a risk factor for the occurrence of choledocholithiasis^52^, and we therefore believe that the alcohol metabolism disorder of biliary microflora plays an important role in the occurrence of choledocholithiasis.

We analyzed the bile acid composition of the two groups using LC–MS. In the choledocholithiasis group, the enrichment degree of metabolites was low. We believe that the normal metabolic function of bacteria was impaired due to the disturbance of biliary microecology, which led to a decrease of the enrichment degree of metabolites. We found that beta-muricholic acid was significantly elevated in the gallbladder polyp group. Mouse experiments have shown that beta-muricholic acid prevents the formation of cholesterol gallstones by reducing biliary cholesterol secretion, retarding phase transition of cholesterol, and inhibiting intestinal cholesterol absorption. Beta-muricholic acid enhanced the dissolution rates of cholesterol gallstones by increasing a liquid crystals-containing phase^53^, but whether beta-muricholic acid has the same effect in humans remains to be explored.

In this study, we further explored the difference between gallbladder polyp patients and choledocholithiasis patients in terms of the composition of the biliary tract microbial community and key species, so that we have a new understanding of the role of the biliary tract microecological community in the occurrence and development of choledocholithiasis, and further explored the biliary tract microecology in healthy people. However, there are some limitations to the experiment. We selected bile specimens from patients with asymptomatic gallbladder polyps as controls, which may be the closest specimens to the bile of healthy people, but certain differences are inevitable. Second, because ERCP is an invasive procedure, there is a risk of oral and gastrointestinal contamination of the bile obtained by this method, and despite our best efforts to ensure sterility, we could not completely ensure that the samples were not contaminated. Therefore, better bile extraction methods need to be explored in the future. It is a pity that no animal experiments have been conducted to verify the role of different bacteria in the occurrence of choledocholithiasis. In future studies, we will further clarify the specific role of bacteria in the occurrence of choledocholithiasis through animal experiments.

## Conclusion

This study is the first to compare the biliary microecology in gallbladder polyp patients and patients with choledocholithiasis. And provides a basis for further studies of biliary microecology in healthy populations. The study indicates the biliary microecological disorder is closely related to the formation of choledocholithiasis, which is mainly manifested by changes in the abundance of bacteria, especially *Actinobacteriota* and *Enterococcus*. Equally important is the discovery of the role of bacterial biofilms, the formation of which may affect the health of the biliary tract and lead to the formation of stones. This discovery will guide the prevention and treatment of common bile duct stones in the future.

## Data Availability

The data presented in the study are deposited in the SRA (https://www.ncbi.nlm.nih.gov/sra/) repository, accession number PRJNA1032404.
